# The impact of learning engagement on the subjective well-being of disadvantaged older adults in China

**DOI:** 10.3389/fpubh.2023.1196692

**Published:** 2023-07-18

**Authors:** Yuan Li, Huiqin Shi, Yaya Yuan, Ruirang Zeng, Binggui Bai, Lixin Sun

**Affiliations:** ^1^College of Teacher Education, Ningbo University, Ningbo, China; ^2^School of Digital Commerce, Zhejiang Business Technology Institute, Ningbo, China; ^3^Faculty of Maritime and Transportation, Ningbo University, Ningbo, China; ^4^School of Continuing Education, Wenzhou University, Wenzhou, Zhejiang, China

**Keywords:** disadvantaged groups, learning to engage, subjective well-being, quantitative studies, economic situation, empty nest

## Abstract

Due to social transformation, economic reform, and the advent of an aging society, the number of disadvantaged older adults in China is increasing. The living conditions of the disadvantaged older adult groups determine society’s stability to a certain extent. How to make their lives happier in their old age, promote their subjective well-being, and realize the “enjoyment of the older adults” is of great practical significance in improving social civilization and building a harmonious society.This study uses questionnaires to obtain survey data from the lower counties of Ningbo City, Zhejiang Province, where there is a high concentration of older learners, and used SPSS 27.0 software to process the data. The results indicated the following: the subjective well-being of disadvantaged and non-disadvantaged older adults differed significantly; learning engagement had a significant effect on enhancing the subjective well-being of disadvantaged older adults, and all dimensions of learning engagement had a significant positive relationship with subjective well-being (SWB). Compared to non-disadvantaged older adults, learning engagement had a more significant contribution to the SWB of disadvantaged older adults.

## 1. Introduction

By the end of 2021, there were 260 million individuals aged 60 and above, of which 190 million were aged 65 and above (1). It was predicted that China’s older adult population will exceed 400 million in 2033 and reach a peak of 487 million in 2053, accounting for 34.9% of the total population (2), making China a country that is “old before it is rich and old before it is ready.” The increasing aging population makes the issue of older adults a global and strategic social problem. As China’s social and economic structure continues to adjust and transform, some older adults are disadvantaged in several aspects of social life due to institutional arrangements, redistribution of interests, and their physiological decline, consequently becoming vulnerable (3). Disadvantaged older adults’ state of life determines society’s stability to a certain extent. Consequently, how to make their lives happier in their old age, promote their subjective well-being, and achieve “a happy old age” has become one of the focuses of academic and social attention.

Subjective well-being (SWB) is an essential comprehensive psychological indicator reflecting the quality of an individual’s life, which is a cognitive evaluation and emotional experience of an individual’s life (4). Goal theory suggests that SWB stems from the satisfaction of needs, the achievement of goals, and the realization of self-worth in milestones, all of which reflect individuals’ needs in life. Disadvantaged older adult groups can gain subjective identity through participation in learning activities, with self-concept and self-activity goals, allowing for their senior needs to be satisfied, thus realizing their self-worth and enhancing their SWB (5). Research on vulnerable older adult groups gradually received scholarly attention in recent years. This study takes the relationship between learning participation and SWB of disadvantaged older adults groups in China as the base point. It investigates the current situation of learning participation and SWB of disadvantaged and non-disadvantaged older adult groups and analyzes the differences in SWB between them and the influencing factors to provide reference experiences and educational countermeasures for improving the SWB of disadvantaged older adult groups. In this study, the disadvantaged older adult group is defined as those eligible for empty nesting and have a monthly income of less than 1,000 RMB, using residence status and economic level as measurement criteria.

## 2. Review of past studies

### 2.1. Research related to disadvantaged older adult groups

The term “disadvantaged group” was first mentioned in China by Premier Zhu Rongji at the Fifth Session of the Ninth National People’s Congress in March 2002, and issues related to this group have subsequently received widespread attention from all sectors of society (6). Researchers in China and abroad generally believed that disadvantaged groups were the most oppressed and uncompetitive groups in society, as opposed to powerful or entitled groups. Disadvantaged groups are generally divided into two categories: physically and socially disadvantaged. The former is caused by unavoidable factors, while the latter is predominantly caused by institutional issues (7). While disadvantaged older adults belong to the former group, there is still no precise prescribed standard for the conceptual definition and classification of the disadvantaged older adult group. Kutek (8) argued that older adults who are socially disadvantaged due to certain barriers and lack economic and political resources could be called the vulnerable older adult group. Mu et al. (9) argued that older adults who live alone and are incapacitated to care for themselves are an important part of the disadvantaged population. Chen (10) showed that the characteristics of disadvantaged older adults include low economic security, lack of spiritual comfort, and insufficient social participation.

By comparing the overall situation of disadvantaged and non-disadvantaged groups in Anhui Province, Li et al. (11) found that disadvantaged groups experience more negative emotions due to the long-term lack of companionship and care from family, friends, or children, and their emotional needs are not met. Keliman et al. (12) found that disadvantaged older adult groups had poorer physical and mental health and were more likely to develop mental illnesses such as Alzheimer’s. Drawing on data from the World Values Survey, Cao found significant differences in SWB across social classes, with disadvantaged older adults of lower economic levels having the lowest levels of SWB, experiencing various difficulties in interacting with others, more commonly experiencing psychological disorders, and facing a higher risk of social isolation (13).

Additionally, Sun et al. (14) found that a significant proportion of vulnerable older adults subscribe to the view that participation in learning is an adolescent and childhood task. They believe that older adults are not suited to continue learning due to organic decline, neither considering the possibility of learning participation nor recognizing the unique significance of learning in later life. This hesitancy reflects this group’s misconceptions and negative attitudes toward learning participation. Participation in learning activity courses still incur financial cost, which is undoubtedly a significant expense for the disadvantaged older adult group, as they are already less financially resilient. Therefore, the cost factor may also be one of the reasons why they give up learning.

Accordingly, Hypothesis 1 was proposed as follow.

*Hypothesis 1*: Disadvantaged older adults have lower physical and mental health and well-being and non-participation in learning activities than non-disadvantaged older adults.

### 2.2. Study on the factors influencing the subjective well-being of disadvantaged older adult groups

Combining the results of domestic and international studies, the factors influencing the SWB of disadvantaged older adults can be divided into three main categories: self, family, and social. First, self-factors are mainly examined from the perspectives of gender, physical condition, and economic level. The quantitative results of Li et al. (15) showed that disadvantaged female older adults have significantly lower SWB than men due to their longer life expectancy and higher widowhood rate. A recent national study has demonstrated that health status is one of the significant factors affecting the subjective well-being of vulnerable older people (16). Disadvantaged older adults are more likely to experience anxiety and fear due to their health status, and physical well-being affects their mood to a greater extent than non-disadvantaged older adults (9). The economic level currently remains the most critical factor affecting the SWB of older people in China. Most disadvantaged older people have poor income status and lack livelihood security, leading to a significant lack of SWB (17). Second, family factors mainly refer to marital status. Related studies confirmed that the SWB of older adults with spouses is higher than those without spouses. Yetter (18) found that marital status has a significant effect on disadvantaged older male adults and that disadvantaged older male adults who live with their spouses are less likely to die unexpectedly and are more satisfied with their lives.

Additionally, Mui and Burnette (19) argued that older disadvantaged female adults could lose confidence in life and develop negative emotions such as loneliness and emptiness due to widowhood. Finally, social factors include external elements such as life support and environment. Meehan (20) pointed out that there is a positive correlation between social support and individuals’ positive emotions; the more social support older adults receive, the more secure their quality of life will be and the higher their SWB. Maintaining a high level of life satisfaction is maintaining solid relationships with family and friends, and the association between social interactions and SWB in older adults cannot be ignored (21). In addition, different social environments may also affect the SWB of older adults. Some studies have shown that rural areas have a more substantial negative impact on disadvantaged older adults’ SWB than those living in urban areas due to poorer economic conditions and activity facilities, resulting in lower levels of social participation.

### 2.3. Study on the impact of learning participation on the subjective well-being of disadvantaged older adults

Throughout domestic and international studies, few have directly explored the relationship between learning participation and the SWB of disadvantaged older adults. Most studies have viewed physical and mental health, quality of life in old age, and social practice participation as perspectives to verify the influence of learning on SWB in old age. As stated in Erikson’s self-development theory, individuals mainly face the developmental dilemmas of physical aging, role transformation, and self-transcendence in old age. These dilemmas are the main contributors affecting older adults’ sense of achievement, value, and well-being.

Disadvantaged older adults’ health is in crisis as various organs and functions of the body gradually deteriorate with age. Coupled with being financially disadvantaged and unable to afford physical care, it eventually leads to a decline in mental health, which reduces life satisfaction and happiness. The World Organization for Economic Cooperation and Development (WECD) has found that mental decline in older adults can be delayed through educational training, which through learning sustains brain power activation and promotes the active aging of individuals (22). A study by Gu and Wang (23) scientifically showed that appropriate geriatric learning could assist vulnerable older adult groups in preventing common diseases. Simultaneously, knowledge of relevant mental health is beneficial for rationally coping with and resolving stress and destructive emotions and maintaining a positive attitude toward life, thus reducing the risk of psychological depression in older adults. Feng et al. (24) argued that old age is a period of physiological decline entailing role changes and loss. Additionally, losing the original role leads to the vulnerable older adult group facing role conflict when retiring from the social production field. At the same time, the departure of children causes the loss of emotional communication and the original role support as parents in the family, which hinders the role adjustment after retirement. This change affects the acquisition of happiness to some extent. Shepherd (25) used the formula of L (speed of learning) > C (speed of change) to present the relationship between learning and social adjustment, pointing out that by adapting to new roles and social development, older adults need to acquire new wisdom and skills. The acquisition of new wisdom and skills can only be achieved through learning. Boulton-Lewis and Li (26) found that participation in various educational activities could help disadvantaged older adults build more effective interpersonal relationships and psychological support systems, consequently contributing to their emotional well-being by gaining more care and help.

Learning to participate is one of the most important ways to promote social participation among the disadvantaged older adult population, not only to slow down the decline of their intellectual abilities but also to assist them in adapting to new roles and consequently increasing their motivation to participate in society. Additionally, it can help maintain normal interpersonal relationships, strengthen interactions with other groups and society as well as emotional attachment, develop positive emotions and avoid negative emotions. Finally, learning to participate can help disadvantaged older adults obtain an enjoyable old age career and enhance SWB. Accordingly, Hypothesis 2 and Hypothesis 3 are proposed as follows:

*Hypothesis 2*: Learning engagement effectively increases the subjective well-being of vulnerable older adults.

*Hypothesis 3*: Learning engagement has a higher positive effect on the subjective well-being of disadvantaged older adults than non-disadvantaged older adults.

## 3. Materials and methods

### 3.1. Sample selection

To further reveal the influence mechanism of learning participation on the SWB of disadvantaged older adults, this study was conducted in 2022 using a questionnaire survey method with a random sample of older adult groups aged 50 and above who were involved in older adult learning in the lower districts and counties of Ningbo, Zhejiang Province, where the concentration of older adult learners was high. Random distribution of electronic questionnaires to the above older adult groups using educational institutions such as community colleges and adult schools (based on domicile) as a platform. The international standard for classifying the age of the older adult is 60 years old, and the relevant policies and regulations in China also stipulate that the older adult are citizens who have reached the age of 60 or above; some people think that the older adult are retired people who have reached the age of 55 and are no longer engaged in social work. In contrast, this study uses age chronology as the defining criterion. The first person to define older people in terms of age chronology was the Swedish scholar Sandbar, who used the age limit of 50 years to delineate older people. Based on the actual context of the study and the requirements for enrolment in senior schools, this study refers to older individuals as seniors who are 60 years of age or older for males and 50 years of age or older for females and who can normally participate in learning activities in senior education institutions. The inclusion criteria and study objectives were explained to all participants and consent for participation was obtained prior to the start of the study. A total of 2036 questionnaires were collected, and after the screening, 2007 valid questionnaires remained, the sample size was calculated using the empirical formula method: the valid questionnaire return rate was as high as 98.6%, the non-response rate was as low as 1.4% and the sample produced less bias, therefore, the data is sufficiently representative and valid. The final sample composition was 1,216 (60.6%) for the disadvantaged older adult group and 791 (39.4%) for the non-disadvantaged older adult group (Table 1).

**Table 1:** Statistics of demographic variables (*n* = 2007).

	Disadvantage	Non-disadvantaged	Overall
Total individuals	1,216 (60.6%)	791 (39.4%)	2007
Age (in years)			
50–59	458 (22.8%)	490 (24.4%)	948 (47.2%)
60–69	541 (27.0%)	244 (12.1%)	785 (39.1%)
70–79	205 (10.2%)	57 (2.8%)	262 (13.1%)
over 80	12 (0.6%)	0 (0.0%)	12 (0.6%)
Gender			
Male	187 (9.3%)	77 (3.8%)	264 (13.2%)
Female	1,029 (51.3%)	714 (35.6%)	1743 (86.8%)
Marital Status			
Married	1,104 (55.0%)	723 (36.0%)	1827 (91.0%)
Single	112 (5.6%)	68 (3.4%)	180 (9.0%)
Monthly income			
Under ¥1,000	95 (4.7%)	0 (0.0%)	95 (4.7%)
¥1,000–¥2,000	282 (14.1%)	218 (10.9%)	500 (24.9%)
¥2,000–¥4,000	457 (22.8%)	328 (16.3%)	785 (39.1%)
Over ¥4,000	382 (19.0%)	245 (12.2%)	627 (31.2%)
Education level			
Less than junior middle school	119 (5.9%)	58 (2.9%)	177 (8.8%)
Junior high school	509 (25.4%)	327 (16.3%)	836 (41.7%)
High school(including technical secondary school and professional high school)	403 (20.1%)	294 (14.6%)	697 (34.7%)
Junior college	123 (6.1%)	80 (4.0%)	203 (10.1%)
College/Bachelor’s degree or above	62 (3.1%)	32 (1.6%)	94 (4.7%)
Living conditions			
Living alone (empty nest)	128 (6.4%)	0 (0.0%)	128 (6.4%)
Living with partner only (empty nest)	1,056 (52.6%)	0 (0.0%)	1,056 (52.6%)
Living with children (non-empty nester)	11 (0.5%)	170 (8.5%)	181 (9.0%)
Living with a partner and children (non-empty nester)	21 (1.0%)	618 (30.8%)	639 (31.8%)
Living in a residential care facility (non-empty nest)	0 (0.0%)	3 (0.1%)	3 (0.1%)
Type of household registration			
Rural	411 (20.5%)	362 (18%)	773 (38.5%)
Town	805 (40.1%)	429 (21.4%)	1,234 (61.5%)

### 3.2. Variable definition and coding

This paper uses the Questionnaire on Older People’s Participation in Older People’s Education and Subjective Well-being as the basis of data research. The questionnaire includes three sections: basic information about older people, learning participation, and subjective well-being. The homogeneity reliability of the questionnaire was 0.91, and the coefficients of each dimension and the total scale were between 0.80 and 0.93. The combined validity of all the variables was greater than 0.7, the average variance extracted (AVE) was greater than 0.5, and the square root of AVE was greater than the correlation coefficient between the generic dimension and other dimensions. These results indicate that the total scale’s reliability meets the study’s requirements.

The basic information about the older adults refers to demographic variables, including individual characteristics and household characteristics: individual characteristics include age, gender, education, monthly income, etc. The household characteristics variables are household registration, marital status, residence status, etc. Older people’s participation in learning is the dependent variable in this paper and consists of three dimensions: older learners’ commitment to learning, learning atmosphere, and learning experience. Thirteen questions were asked about participation in learning. After standardizing the above data, Cronbach’s alpha for the explanatory variables was 0.854, indicating good overall reliability and statistical significance. The results of the validation factor analysis indicated a good model fit: χ2/df = 1.762, CFI = 0.99, TLI = 0.986, GFI = 0.972, and RMSEA = 0.041, indicating good structural validity of the scale.

The dependent variable in this study is subjective well-being of the older adult. In the design of the scale, reference was made to the “Chinese Urban Residents’ Subjective Well-being Scale (short version),” and on the basis of the original structure, dimensions and scoring method of this questionnaire, the formulation and content of some questions of the original questionnaire were adjusted accordingly, taking into account the field survey. The subjective well-being of older people in this study is categorized into three dimensions: physical and mental health experience, adaptation satisfaction experience and self-development experience, comprising a total of 10 items on a 6-point scale ranging from ‘1 - strongly disagree’ to ‘6 - strongly agree’, with higher scores indicating a higher level of subjective well-being.

After standardization, the reliability of the explanatory variables was tested, and Cronbach’s alpha was 0.882, indicating good overall reliability. The results of the validation factor analysis indicated a good model fit: χ^2^/df = 1.157, CFI = 0.984, TLI = 0.997, GFI = 0.997, and MSEA = 0.019, indicating good construct validity of the scale. The specific coding and assignment criteria for the above demographic, explanatory, and explained variables are detailed in Table 2.

**Table 2:** Variable codes and explanations.

		Value	Variable Explanation
Age		1–4	1 = 50–59;2 = 60–69;3 = 70–79;4 = over 80
Gender		1–2	1 = Male; 2 = Female
Marital Status		1–2	1 = Married; 2 = Single
Type of household registration		1–2	1 = Rural; 2 = Town
Living conditions		1–5	1 = Living alone (empty nest) 2 = Living with partner only (empty nest) 3 = Living with children (non-empty nester) 4 = Living with partner and children (non-empty nester) 5 = Living in a residential care facility (non-empty nester)
Monthly income		1–4	1 = Under ¥1,000; 2 = ¥1,000–¥2,000; 3 = ¥2,000–¥4,000; 4 = Over ¥4,000
Education level		1–5	1 = Less than junior middle school; 2 = Junior high school; 3 = High school (includes technical secondary school and professional high school); 4 = Junior college; 5 = College/Bachelor’s degree or above
SWB	Physical and mental health experience	1–6	From 1 (strongly disagree) to 6 (strongly agree). The higher the score, the higher the subjective well-being of the older people.
	Adaptation satisfaction experience		
	Self-development experiences		
Older adult learning	Learning investment	1–6	From 1 (strongly disagree) to 6 (strongly agree). The higher the score, the higher the level of participation in learning by older people.
	Learning atmosphere		
	Learning experience		

### 3.3. Data analyses

SPSS 27.0 was utilized for all analyses.

1) Descriptive statistics and variance tests were used to analyze the differences in demographic variables in the SWB and learning participation dimensions of disadvantaged older adults.
2) An independent sample t-test was conducted to determine whether there was a significant difference between the SWB and learning engagement dimensions of disadvantaged and non-disadvantaged older adults.
3) Correlation analysis was used to identify the relationship between learning engagement and the SWB of vulnerable older adults.
4) Based on the correlation analysis, a stratified regression method was used to examine the mechanism of the effect of learning participation on the SWB of the disadvantaged older adult group and to discover the specific differences in the effect of learning participation on enhancing the SWB of the disadvantaged and non-disadvantaged older adult groups.

## 4. Results

### 4.1. Descriptive statistical analysis and test of variance

The study data showed (Table 3) that in the SWB dimension, there were highly significant differences in the SWB of the older adult groups by gender, age, monthly income, education, and household registration. Among them, the happiness level of the female group was higher than that of the male group. Regarding the age dimension, the subjective happiness of the older adult group aged 80 and above was the lowest; regarding the monthly income dimension, the subjective happiness of the older adult group earning between 1,000 and 2,000 RMB was the highest; regarding the household registration factor, rural areas were higher than urban areas. Additionally, there were significant differences in SWB among older adult groups with different residence statuses, and the SWB of non-empty nesting older adult groups was significantly higher than that of empty nesting older adult groups. In comparison, no significant difference was identified in the SWB of older adult groups with different marital statuses.

**Table 3:** Descriptive statistics and difference test results of variables.

	SWB	Learning atmosphere	Learning Experience	Learning investment
Single	5.52 ± 0.78	5.52 ± 0.74	5.52 ± 0.76	3.80 ± 0.63
Married	5.44 ± 0.90	5.45 ± 0.89	5.47 ± 0.89	3.87 ± 0.70
t	1.07	1.00	0.75	−1.49
Male	5.25 ± 0.95	5.27 ± 0.89	5.27 ± 0.96	3.80 ± 0.66
Female	5.55 ± 0.76	5.55 ± 0.73	5.55 ± 0.74	3.80 ± 0.64
t	−4.82***	−4.78***	4.49***	−0.19
50-59	5.55 ± 0.74	5.57 ± 0.68	5.56 ± 0.71	3.70 ± 0.58
60–69	5.51 ± 0.79	5.51 ± 0.77	5.52 ± 0.78	3.85 ± 0.65
70–79	5.39 ± 0.88	5.39 ± 0.89	5.37 ± 0.89	4.04 ± 0.71
Over 80	4.14 ± 1.25	4.28 ± 1.35	4.07 ± 1.26	3.50 ± 0.82
F	15.22***	14.23***	18.44***	22.89***
Under ¥1,000	5.45 ± 0.96	5.41 ± 1.03	5.41 ± 1.00	3.45 ± 0.67
¥1,000–¥2,000	5.62 ± 0.70	5.58 ± 0.74	5.61 ± 0.69	3.65 ± 0.62
¥2,000–¥4,000	5.50 ± 0.79	5.51 ± 0.73	5.52 ± 0.76	3.78 ± 0.63
Over ¥4,000	5.44 ± 0.83	5.46 ± 0.76	5.44 ± 0.81	4.01 ± 0.61
F	4.86**	2.84*	5.08***	43.53***
Less than junior middle school	5.67 ± 0.74	5.63 ± 0.79	5.63 ± 0.80	3.61 ± 0.69
Junior high school	5.59 ± 0.72	5.58 ± 0.69	5.59 ± 0.69	3.72 ± 0.64
High school (includes technical secondary school and professional high school)	5.50 ± 0.76	5.50 ± 0.75	5.51 ± 0.76	3.89 ± 0.61
Junior college	5.25 ± 0.94	5.27 ± 0.87	5.23 ± 0.92	3.96 ± 0.63
College/Bachelor’s degree or above	5.10 ± 1.08	5.25 ± 0.89	5.20 ± 1.00	3.94 ± 0.53
F	15.76***	11.11***	13.96***	14.78***
Living alone (empty nest)	5.38 ± 0.98	5.40 ± 0.95	5.38 ± 0.96	3.78 ± 0.70
Living with a partner only (empty nest)	5.48 ± 0.83	5.48 ± 0.79	5.49 ± 0.79	3.82 ± 0.64
Living with children (non-empty nester)	5.62 ± 0.68	5.57 ± 0.72	5.61 ± 0.66	3.88 ± 0.63
Living with a partner and children (non- empty nester)	5.55 ± 0.71	5.56 ± 0.66	5.54 ± 0.73	3.75 ± 0.62
Living in a residential care facility (non-empty nester)	6.00 ± 0.00	6.00 ± 0.00	6.00 ± 0.00	3.56 ± 0.77
F	3.02*	2.26	2.47*	2.08
Rural	5.62 ± 0.70	3.65 ± 0.60	5.60 ± 0.69	5.63 ± 0.67
Town	5.44 ± 0.84	3.90 ± 0.65	5.45 ± 0.79	5.44 ± 0.82
t	5.24***	−8.64***	4.56***	5.52***

* *p* ≤ 0.05.

** *p* ≤ 0.01.

*** *p* ≤ 0.001.

In each dimension of older adult learning participation, there were significant differences in the learning atmosphere, learning experience, and learning commitment among older adult groups with different education, age, monthly income, and household registration type. There were no significant differences in learning commitment among senior groups of different gender, and there were differences in learning experience among senior groups with varying residence statuses. Additionally, there were no significant differences in the learning atmosphere, learning experience, and learning commitment among older adults with different marital statuses.

### 4.2. Independent sample *t*-test analysis of learning engagement and subjective well-being of disadvantaged and non-disadvantaged older adults

To further clarify the differences between disadvantaged and non-disadvantaged older adult groups regarding learning participation and SWB, a t-test of independence was conducted on the sample data using SPSS software (Table 4). The study data showed significant differences between disadvantaged and non-disadvantaged older adult groups regarding the learning atmosphere. Additionally, regarding mean values, the disadvantaged older adult group obtained lower scores than the non-disadvantaged older adult group in the learning atmosphere and learning experience dimensions. Furthermore, there were significant differences between these two groups in all three dimensions of SWB, and the disadvantaged older adult group was not as good as the non-disadvantaged older adults group in all three dimensions. Overall, there were significant differences in learning engagement and SWB between the disadvantaged and non-disadvantaged older adult groups. The former group had lower physical and mental health and well-being and performed more negatively in learning activity participation. Consequently, research hypothesis 1 was verified.

**Table 4:** Results of independent samples t-test (*n* = 2007).

Variables		Disadvantage	Non-Disadvantage	*t*	*p*
		M(SD)	M(SD)		
Older adult Learning	Learning investment	3.81 (0.65) 5.48 (0.80) 5.49 (0.81) 5.42 (0.91) 5.46 (0.88) 5.54 (0.80)	3.97 (0.62) 5.55 (0.68) 5.55 (0.72) 5.52 (0.77) 5.55 (0.77) 5.62 (0.67)	0.90	0.37
	Learning atmosphere			−1.97	0.05
	Learning experience			−1.82	0.07
SWB	Physical and mental health experience			−2.59	0.01
	Self-development experiences			−2.23	0.02
	Adaptation to meet the experience			−2.23	0.03

### 4.3. Correlation analysis of learning participation and subjective well-being of disadvantaged older adults

Pearson correlation analysis was conducted (Table 5) to investigate the correlation between learning engagement and the SWB of disadvantaged older adults. The results showed that all dimensions of learning engagement of disadvantaged older adults were positively correlated with physical and mental health experience, self-development experience, adaptation satisfaction experience, and overall SWB. The correlation was significant (*p* < 0.01). Among them, learning investment was lowly correlated with the SWB of disadvantaged older adults, the learning atmosphere was moderately correlated with the SWB of disadvantaged older adults, and learning experience was highly correlated with the SWB of disadvantaged older adults. The analysis indicated that the participation in learning activities of the disadvantaged older adult group could significantly enhance their SWB. Therefore, hypothesis 2 was verified.

**Table 5:** Correlation analysis of the dimensions of subjective well-being of learning participation among vulnerable older adults (*n* = 1,216).

	1	2	3	4	5	6	7
Learning atmosphere	1						
Learning experience	0.848**	1					
Learning investment	0.112**	0.111**	1				
Physical and mental health experiences	0.733**	0.812**	0.122**	1			
Self-development experiences	0.761**	0.854**	0.150**	0.921**	1		
Adaptation to meet the experience	0.785**	0.865**	0.143**	0.894**	0.932**	1	
SWB	0.781**	0.867**	0.142**	0.968**	0.979**	0.967**	1

* *p* ≤ 0.05.

** *p* ≤ 0.01.

*** *p* ≤ 0.001.

### 4.4. Stratified regression analysis of the effect of learning engagement on the subjective well-being of disadvantaged and non-disadvantaged older adults

#### 4.4.1. Multicollinearity test

This study used a hierarchical regression method to explore the effects of learning atmosphere, learning experience, and learning engagement on the SWB of disadvantaged and non-disadvantaged older adults. The problem of multicollinearity needed to be tested before conducting the regression analysis. Multicollinearity means that the high correlation between variables affects the analysis results. The condition for judging multicollinearity is that the variance inflation factor (VIF) is greater than 10.00 while the mean VIF is greater than 1.00. Although the mean VIF (1.67) in this study was slightly greater than 1.00, the VIF of all variables was less than 5.00, indicating no serious multicollinearity problem and that stratified regression could be conducted.

#### 4.4.2. Stratified regression model construction

Based on the above descriptive statistical and correlation analyses, separate stratified regressions were conducted for disadvantaged and non-disadvantaged older adult groups. Model 1 only included learning variables (learning atmosphere, learning experience, and learning investment); model 2 added individual demographic characteristics factors (age, gender and education); and model 3 sequentially added household factors from demographic variables (marital status and household registration type, etc.) These models were chosen to more comprehensively reflect the impact of learning participation on the SWB of disadvantaged and non-disadvantaged older adult groups and its changing trend. The following multiple linear regression model was utilized, where Y referred to the dependent variable (subjective well-being) and X_1_, X_2_, … X_k_ referred to the independent variables (older adult learning participation and its factors):


(1) Yi=β0+β1X1+β2X3...+βiXi+ε

Regression analysis of the total sample of older adults found that the standardized equations for the three multiple linear regressions for the full, disadvantaged, and non-disadvantaged samples were as follows:


$$\begin{array}{l} Y\;\left(\mathrm{full sample}\right)=0.192\times \mathrm{learning atmosphere}+0.714\\ \;\;\;\;\;\;\;\;\;\;\;\;\;\;\;\;\;\;\;\;\;\;\;\;\;\;\;\;\;\;\;\;\;\;\;\;\;\;\;\;\;\;\times \mathrm{learning experience}+0.030\\ \;\;\;\;\;\;\;\;\;\;\;\;\;\;\;\;\;\;\;\;\;\;\;\;\;\;\;\;\;\;\;\;\;\;\;\;\;\;\;\;\;\;\times \mathrm{learning investment} \end{array}$$

$$\begin{array}{l} Y\;\left(\mathrm{disadvantaged sample}\right)=0.166\times \mathrm{learning atmosphere}+0.755\\ \;\;\;\;\;\;\;\;\;\;\;\;\;\;\;\;\;\;\;\;\;\;\;\;\;\;\;\;\;\;\;\;\;\;\;\;\;\;\;\;\;\;\;\;\;\;\;\;\;\;\;\;\;\;\;\;\;\;\;\;\;\;\;\;\;\;\;\;\times \mathrm{learning experience}+0.056\\ \;\;\;\;\;\;\;\;\;\;\;\;\;\;\;\;\;\;\;\;\;\;\;\;\;\;\;\;\;\;\;\;\;\;\;\;\;\;\;\;\;\;\;\;\;\;\;\;\;\;\;\;\;\;\;\;\;\;\;\;\;\;\;\;\;\;\;\;\times \mathrm{learning investment} \end{array}$$

$$Y\;\left(\begin{array}{l} \mathrm{non}\\ -\mathrm{disadvantaged sample} \end{array}\right)=\begin{array}{c} 0.255\times \mathrm{learning atmosphere}+0.651\\ \times \;\mathrm{learning experience}+0.044\\ \;\times \mathrm{learning investment} \end{array}$$

The results of model 1 indicated that learning atmosphere, learning experience, and learning investment all have a significant positive effect on the SWB of overall older adults (*F* = 1926.256, *p* < 0. 001) with R-square = 0.743 (which explains 74.3% of the variance probability of SWB). Based on the comparison of the regression results of SWB of the disadvantaged and non-disadvantaged older adult group samples, the two influencing factors of learning investment and learning experience had higher positive effects on SWB in the disadvantaged older adult group than that in the non-disadvantaged older adult group. Only one factor, learning atmosphere, had higher positive effects on SWB in the non-disadvantaged older adult group than in the disadvantaged older adult group. In a comprehensive analysis, the positive effect of learning engagement on the SWB of the disadvantaged older adult group was higher than that of the non-disadvantaged older adult group.

To further test the hypothesis, the degree of influence of learning engagement on the overall SWB of the older adult groups increased with the addition of variables related to individual characteristics and family factors in models 2 and 3. In contrast, the degree of influence of the learning atmosphere and learning experience decreased but remained significantly positive. In the sample of the disadvantaged older adult group, it was shown that after including individual factors, the influence of learning investment on their SWB increased significantly. In contrast, the influence of the learning atmosphere and learning experience decreased. With the intervention of family factors, the influence of the learning atmosphere and learning investment increased, while the influence of the learning experience decreased. The regression results of the SWB of the non-disadvantaged older adults group showed that the effect of all dimensions of learning participation on their SWB decreased and that learning engagement had a negative effect on them after adding individual factors; after including family factors, the effect of the learning atmosphere and learning experience on their SWB increased, while the effect of learning investment decreased. Although the effect of learning engagement on SWB in disadvantaged and non-disadvantaged older adult groups changed by different degrees after adding individual characteristics and family factor variables, the positive effects of learning experience and learning investment on SWB in disadvantaged older adult groups remained higher than those in non-disadvantaged older adult groups.

The results showed that before and after adding individual and family factors, the positive effect of the learning atmosphere and learning experience on the SWB of both disadvantaged and non-disadvantaged older adult groups was more pronounced and more thoroughly explained their SWB. Additionally, the coefficients of learning experience and learning investment on the SWB of the disadvantaged older adult group were higher than those of the non-disadvantaged older adult group. Moreover, learning investment had a negative effect on the SWB of the non-disadvantaged older adult group. The combined model 1, 1a, 1b; 2, 2a, 2b; 3, 3a, 3b analysis indicated that the positive effect of learning participation on the SWB of the disadvantaged older adult group was more pronounced. Therefore, hypothesis 3 was supported.

#### 4.4.3. Robustness test

To further ensure the reliability of the findings obtained from the above hierarchical regression, this study adopted a model change approach to replace the used hierarchical regression model with an ordinary least squares (OLS) regression model, drawing on the robustness testing method conducted by Wu et al. (27). The results of the OLS regression model show that the positive effect of learning engagement on the subjective well-being of disadvantaged older people is more pronounced and is largely similar to the overall picture reported in Table 6. Therefore, the results of the stratified regression are robust and the findings are more reliable.

**Table 6:** Regression results of the effect of learning engagement on subjective well-being of older adults (*n* = 2007).

	Full sample			Sample of disadvantaged groups			Sample of non-disadvantaged groups		
	Model 1	Model 1a	Model 1b	Model 2	Model 2a	Model 2b	Model 3	Model 3a	Model 3b
Learning atmosphere	0.192*** (8.928)	0.189*** (8.786)	0.188*** (8.756)	0.165*** (5.981)	0.162*** (5.907)	0.163*** (5.952)	0.225*** (6.526)	0.225*** (6.489)	0.224*** (6.463)
Learning experience	0.714*** (33.816)	0.707*** (33.434)	0.707*** (33.397)	0.755*** (27.520)	0.745*** (27.102)	0.742*** (26.947)	0.651*** (19.865)	0.649*** (19.665)	0.650*** (19.737)
Learning investment	0.030* (2.158)	0.041** (2.838)	0.042** (2.862)	0.056** (3.091)	0.072*** (3.843)	0.075*** (3.941)	−0.010*** (−0.472)	−0.011*** (−0.469)	−0.011*** (−0.486)
Age		−0.010 (−0.728)	−0.005 (−0.371)		−0.014 (−0.818)	−0.008 (−0.459)		0.014 (0.590)	0.013 (0.502)
Gender		0.031 (1.116)	0.037 (1.290)		0.023 (0.670)	0.029 (0.852)		0.029 (0.575)	0.032 (0.620)
Educational level		−0.034** (−3.469)	−0.034** (−3.212)		−0.053*** (−4.178)	−0.047** (−3.465)		−0.005 (−0.297)	−0.015 (−0.858)
Marital Status			−0.042 (−1.319)			−0.022 (−0.529)			−0.059 (−1.132)
Type of household registration			0.006 (−0.291)			−0.37 (−1.387)			0.043 (1.404)
N	2007	2007	2007	1,216	1,216	1,216	791	791	791
R2	0.743	0.745	0.745	0.761	0.764	0.764	0.706	0.705	0.705
F	1926.256***	971.458***	728.764***	1289.867***	656.941***	493.068***	632.849***	315.593***	237.429***

* *p* ≤ 0.05.

** *p* ≤ 0.01.

*** *p* ≤ 0.001.

## 5. Research conclusion and discussion

### 5.1. Discussion

First, there were significant differences in SWB between disadvantaged and non-disadvantaged older adult groups, and disadvantaged older adult groups exhibited more negative behaviors in learning participation. In this study, the disadvantaged older adult group mainly referred to those who are empty nesters and have poor economic levels. Regarding this group, three aspects of disease medical care, life care, and mental health care are the main problems they face (28). Most disadvantaged older adults lack their relatives’ companionship and social support, have low life satisfaction, and are more lonely than other non-disadvantaged older adults (29). Regarding material and economic conditions, this group has low disposable income in their old age, and they need to rely on their children, relatives, and friends to support them in their living and financial affairs.

Regarding learning, most disadvantaged older adults lack self-confidence, believe that their learning ability is inferior to others, have a weak self-affirmation, and want to spend little or no money on learning during old age. The proportion of disadvantaged older adults in the state of “watching more and participating less” is as high as 91.6% (30). This study found that community courses are open to disadvantaged groups for free. However, the number of sessions is small; simultaneously, the scope of community courses is limited, and disadvantaged groups far from the community college may have to give up the training due to various reasons such as transportation and time (31). Due to the impact of modern technology, many urban older adult disadvantaged groups have a strong interest in computer, digital, network, communication, and other computer technology knowledge. In contrast, the current provision of older adult education content is mostly based on traditional courses such as literature, calligraphy, and cooking. For older adult groups, it is challenging to keep up with social development and changes in their learning interests. Therefore, the number of teaching courses offered, the radiation range, and the matching of teaching contents affect disadvantaged older adult groups’ enthusiasm to participate in learning activities.

In addition, this study indicated that there are significant gender differences in SWB, with higher scores for older female adults. These differences are related to the traditional Chinese family model of “male domination and female domination, “where Chinese women have taken on the role of family caregivers and are less socially engaged. When they enter the field of study in later life and engage in learning activities, the need to reconstruct their social circle and are more likely to gain emotional support by making new friends and are, consequently, more likely to have higher SWB by making new friends for emotional support (32). Compared to other age groups, those aged 80 years and older have the worst outcome in all three areas of SWB and learning engagement. Additionally, poor physical and mental health due to advanced age should be critically examined (33).

Second, learning engagement has a significant role in enhancing the SWB of the disadvantaged older adult group, and all dimensions of learning engagement have a significant positive relationship with SWB. Learning engagement includes three dimensions: learning time investment, monetary investment, and the number of courses for disadvantaged older adults. In defining returns to education, Schultz (34) pointed out that individuals’ perceptions of well-being can be achieved through non-monetary returns such as self-efficacy, social capital, and the perceived social status of the educated. Monetary returns, such as course investments, are financially burdensome for disadvantaged older adults. However, once invested, they are more likely to cherish the hard-earned learning opportunities. In this way, they are motivated to recognize gradually the value of the learning content. After participating in educational activities, they are physically and mentally happy, and contribute to the harmonious development of family and social relationships (35). The measurement of the learning atmosphere element mainly involves the perception and evaluation of the teacher’s teaching ability, and the degree of rapport between teacher and students. Social support theory, based on social connection, strongly argues for the correlation between the learning atmosphere and SWB. Through social connection, individuals can reduce psychological contingency reactions, relieve mental tension, and improve social adjustment. Learning experiences focus on perceptions of older learners’ interest in learning, their initiative in learning, the degree of focus on learning, and the importance of learning. Cathie’s research shows that adults’ participation in lifelong learning positively impacts their physical and mental health and that disadvantaged older adults who focus on learning give less thought to what was once unpleasant, such as engaging in learning activities with explicit learning. Suppose they have a clear interest and need to learn. In that case, they will be motivated to overcome difficulties in the learning process and complete the learning activities, increasing their positive emotional experience and, consequently, their subjective well-being (36).

This study also found that monthly income influenced learning engagement, with older adults with a monthly income of $4,000 or more having the highest learning engagement. The lower the education level, the better the learning experience of older adults, most likely due to the lack of previous education, making them value the existing educational opportunities and the unique experience of the learning process (37). Since urban areas have better learning facilities and activities than rural areas and have increased access to learning activities, urban older adults have a better learning environment than rural areas (38).

Third, compared to the non-disadvantaged older adult group, learning engagement significantly contributes to the SWB of the disadvantaged older adult group. After controlling for the variables of individual factors (gender, age, monthly income, and education level) and family factors (household registration, marital status, and residence status) in a stratified regression, the comparison revealed that the two dimensions of learning engagement, learning experience, and learning investment more thoroughly explained the SWB of the disadvantaged older adult group. First, regarding the learning experience, compared to non-disadvantaged older adults, disadvantaged individuals lack children’s care and emotional comfort in their daily lives and thus have more pronounced characteristics of “empty nest syndrome” (39). The learning experience, as emotional and behavioral support, allows them to experience the friendliness and rapport of their instructors and peers, establish new social relationships, and alleviate negative emotions such as anxiety and loneliness by obtaining spiritual help. Therefore, in the reality that the “empty nest characteristic” is more serious, the disadvantaged older adult group has a higher positive effect on enhancing SWB than the non-disadvantaged older adult group.

Material and spiritual conditions are essential criteria for measuring SWB and life satisfaction. The poor economic foundation is another characteristic distinguishing the disadvantaged older adult group from the non-disadvantaged older adult group. When the former cannot change their material conditions to obtain life satisfaction, enriching their spiritual life is undoubtedly a critical way to enhance their SWB. Learning is also a significant spiritual food in an individual’s life, in which they can rediscover their values and have new spiritual pursuits to enrich their lives (40). Therefore, based on the above, the disadvantaged older adult group focusing on learning activities will be more likely to experience the pleasure and happiness brought by learning activities. Second, on the learning engagement dimension, many disadvantaged older adults report having to take care of their children and grandchildren after retirement and having little extra time to engage in other learning activities.

On the contrary, the non-disadvantaged older adult group has more energy to focus on learning because they do not live with their children and have a clear understanding and definition of essential goals. Disadvantaged older adults entering retirement need a clear role to play. The influence of spiritual and cultural activities such as literary creation enables them to re-choose, define, and adapt to their new social roles and live their old age peacefully. As a result, their subjective sense of well-being will also be higher than that of non-disadvantaged older adult groups.

### 5.2. Limitations and future directions

Due to the limitations of disciplinary perspective and research design, some shortcomings still need to be addressed in this study. First, the study sample was concentrated in the lower counties of Ningbo city in Zhejiang province; the study needs more breadth and representativeness of the disadvantaged older adult groups nationwide. The sample could be expanded to extend the findings of this study to a larger scale by reaching out to a broader group of older individuals in rural and inland areas. Second, this study adopted a quantitative research method; while the SWB of the research topic has a complex psychological mechanism, questionnaires and other methods are more reflective of statistical results and symbolic characteristics. Therefore, an interview and narrative method will be considered a follow-up to interpret the research questions in more depth. Thirdly, in the age structure of the sample in this paper, the age group of 50–69 has the largest sample size, while the two age groups of 70–79, 80, and above have a relatively small sample size; therefore, the sample representativeness of these two age groups is slightly under-represented, and subsequent consideration will be given to expanding the sample size by increasing the recruitment range or time of the sample in these age groups. Fourthly, this paper uses an online format to distribute the electronic questionnaire, which requires a certain level of digital literacy from the participants, and this may affect the structure of the sample. Therefore, in subsequent studies, offline questionnaires will be used as far as possible.

### 5.3. Concluding remark

The innovation of this paper is mainly reflected in the following aspects: first, the research object has unique characteristics. In November 2015, it could be seen, from the document “Education 2030 Framework for Action: Ensuring Inclusive, Equitable and Quality Education for All for Lifelong Learning” promulgated by UNESCO, that the living status of disadvantaged groups is generally paid international attention (41), disadvantaged older adult groups belong to the physiologically disadvantaged group, and the intensification of population aging requires more attention on disadvantaged older adult groups. Studying the current situation of disadvantaged older adult groups can help to improve their quality of life and survival and development capacity while guaranteeing that the disadvantaged older adult groups enjoy the fruits of social development. Thus, it is significant to promote the active participation of disadvantaged older individuals in social integration. Second, the research content is relatively new. Most previous studies focused on the health and survival of disadvantaged older adult groups and considered improving their quality of life through economic and material social support. However, few studies directly related learning participation to SWB to enrich their spiritual life in old age. According to the survey data, 69.9% of disadvantaged older adults strongly desire to learn (42). Therefore, this study examined the learning participation and SWB of disadvantaged older adults to draw society’s attention to the learning of disadvantaged older adults and to provide a reference and theoretical basis for the relevant departments to formulate policies.

## Data availability statement

The original contributions presented in the study are included in the article/supplementary material, further inquiries can be directed to the corresponding authors.

## Ethics statement

The studies involving human participants were reviewed and approved by Medical Ethics Committee of Ningbo University. The patients/participants provided their written informed consent to participate in this study.

## Author contributions

YL conceived the framework of the article and the research design, and wrote the entire manuscript. HS conducted the data analysis and participated in the design of the article framework and the implementation of the research process. YY and RZ designed the questionnaire and participated in the sample data collection. BB was responsible for the submission and revision of the article. LS managed and coordinated the planning and implementation of the research activities. All authors contributed to the article and approved the submitted version.

## Funding

This study was funded by “A Study on the Path and Mechanism of Collaborative Governance of Community Education Stakeholders in China” (No: BKA200235).

## Conflict of interest

The authors declare that the research was conducted in the absence of any commercial or financial relationships that could be construed as a potential conflict of interest.

## Publisher’s note

All claims expressed in this article are solely those of the authors and do not necessarily represent those of their affiliated organizations, or those of the publisher, the editors and the reviewers. Any product that may be evaluated in this article, or claim that may be made by its manufacturer, is not guaranteed or endorsed by the publisher.
